# Problematic Internet Use among Adults: A Cross-Cultural Study in 15 Countries

**DOI:** 10.3390/jcm12031027

**Published:** 2023-01-29

**Authors:** Olatz Lopez-Fernandez, Lucia Romo, Laurence Kern, Amélie Rousseau, Bernadeta Lelonek-Kuleta, Joanna Chwaszcz, Niko Männikkö, Hans-Jürgen Rumpf, Anja Bischof, Orsolya Király, Ann-Kathrin Gässler, Pierluigi Graziani, Maria Kääriäinen, Nils Inge Landrø, Juan José Zacarés, Mariano Chóliz, Magali Dufour, Lucien Rochat, Daniele Zullino, Sophia Achab, Zsolt Demetrovics, Mark D. Griffiths, Daria J. Kuss

**Affiliations:** 1 Faculty of Psychology, Campus de Somosaguas, Universidad Complutense de Madrid, Ctra. de Húmera s/n, Pozuelo de Alarcón, 28223 Madrid, Spain; 2 Universidad Francisco de Vitoria, Psychology, Carretera de Pozuelo a Majadahonda km. 1.800, Pozuelo de Alarcón, 28223 Madrid, Spain; 3 Centro de Estudios Universitarios Cardenal Cisneros, University College, Department of Psychology, Calle del General Díaz Porlier 58, 28006 Madrid, Spain; 4 Hospital Universitario Fundación Jiménez Díaz, Servicio de Psiquiatría, Avda. Reyes Católicos 2, 28040 Madrid, Spain; 5 EA 4430 CLIPSYD Université Paris Nanterre, 92000 Paris, France; 6 Hôpital Universitaire Raymond Poincaré, AP-HP Garches, CESP, U1018 INSERM UPS UVSQ 2, 92380 Garches, France; 7 EA 2931, Centre de Recherches sur le Sport et le Mouvement (CESRM), Université Paris Nanterre, 92000 Nanterre, France; 8 Laboratoire EA 4430, CLIPSYD Paris Nanterre, UPN, Université Paris Nanterre, 92000 Nanterre, France; 9 Laboratoire APEMAC EA 4360, Université de Lorraine, 57000 Metz, France; 10 Psychology Department, Centre d’Etudes et Recherches en Psychopathologie et Psychologie de la Santé (EA7411), Université Toulouse Jean Jaurès, 31058 Toulouse, France; 11 Department of Psychology, The John Paul II Catholic University of Lublin, al. Racławickie 14, 20-950 Lublin, Poland; 12 Research Unit of Health Science and Technology, Faculty of Medicine, University of Oulu, P.O. Box 5000, 90014 Oulu, Finland; 13 School of Health and Social Care, Oulu University of Applied Sciences, 90570 Oulu, Finland; 14 Department for Psychiatry and Psychotherapy, University of Lübeck, 23538 Lübeck, Germany; 15 Institute of Psychology, ELTE Eötvös Loránd University, 1064 Budapest, Hungary; 16 An der Questenhorst 7, 30173 Hannover, Germany, Psychotherapeutical Office Pilgramm, Karmarschstr. 44, 30159 Hannover, Germany; 17 LPS EA 849, Aix-Marseille University, 13007 Marseille, France; 18 Psychologie, Langues, Lettres et Histoire Département, University of Nîmes, 30000 Nîmes, France; 19 Medical Research Center Oulu, Oulu University Hospital, University of Oulu, 90220 Oulu, Finland; 20 Clinical Neuroscience Research Group, Department of Psychology, University of Oslo, Forskningsveien 3A, 0373 Oslo, Norway; 21 Department of Developmental and Educational Psychology, University of Valencia, 46010 Valencia, Spain; 22 Department of Basic Psychology, University of Valencia, 46010 Valencia, Spain; 23 Département de Psychologie, Université du Québec à Montréal, 514-987-3000 Poste 4553, Montréal, QC H2L 2C4, Canada; 24 Specialized Facility in Behavioral Addiction ReConnecte, Department of Mental Health and Psychiatry, University Hospitals of Geneva, Rue du Grand-Pré 70 c, 1202 Geneva, Switzerland; 25 Psychological and Sociological Research and Training Unit, Department of Psychiatry, University of Geneva, 1205 Geneva, Switzerland; 26 Centre of Excellence in Responsible Gaming, University of Gibraltar, Gibraltar GX11 1AA, Gibraltar; 27 International Gaming Research Unit, Psychology Department, Nottingham Trent University, Burton Street, Nottingham NG1 4FQ, UK; 28 Cyberpsychology Research Group, Psychology Department, Nottingham Trent University, Nottingham NG1 4FQ, UK

**Keywords:** internet addiction, problematic internet use, problematic gaming, problematic social networking, problematic gambling, problematic online sex, problematic online shopping, cross-cultural research, psychopathology, impulsivity

## Abstract

Background: The present study compared adult usage patterns of online activities, the frequency rate of problematic internet use (PIU), and risk factors (including the psychopathology associated with PIU, i.e., distress and impulsivity) among adults in 15 countries from Europe, America, and Asia. Methods: A total of 5130 adults from Belgium, Finland, Germany, Italy, Spain, France, Switzerland, Hungary, Poland, UK, Norway, Peru, Canada, US, and Indonesia completed an online survey assessing PIU and a number of psychological variables (i.e., depression, anxiety, stress, and impulsivity). The sample included more females, with a mean age of 24.71 years (SD = 8.70). Results: PIU was slightly lower in European countries (rates ranged from 1.1% in Finland to 10.1% in the UK, compared to 2.9% in Canada and 10.4% in the US). There were differences in specific PIU rates (e.g., problematic gaming ranged from 0.4% in Poland to 4.7% in Indonesia). Regression analyses showed that PIU was predicted by problematic social networking and gaming, lack of perseverance, positive urgency, and depression. Conclusions: The differences in PIU between countries were significant for those between continental regions (Europe versus non-European countries). One of the most interesting findings is that the specific PIU risks were generally low compared to contemporary literature. However, higher levels of PIU were present in countries outside of Europe, although intra-European differences existed.

## 1. Introduction

According to the International Telecommunication Union (ITU) [1], there are 4.9 billion internet users, indicating that men and women use it comparably (89% men and 88% women). However, there is a generation gap in internet use because 71% of young people (i.e., 15–24 year-olds) are internet users, compared to 57% among other age groups. Geographically, Europe is the region with the highest internet access (87%). Given the widespread internet access, it is important to cross-culturally assess problematic internet use (PIU) and concurrent mental health issues. PIU has been found to be comorbid with other psychopathologies [2,3], which suggests common vulnerabilities [4,5]. However, there is insufficient scientific evidence regarding the association of contemporary PIU problems among adult populations globally. Therefore, the present study examined PIU and whether PIU was associated with personality and psychopathological variables at both the country and global levels.

In terms of published peer-reviewed studies examining PIU, the main topics include (ordered by number of scientific papers published until 2017 [6]): (i) PIU and its association with mental health problems, (ii) PIU and depression among undergraduates, and (iii) PIU prevalence rate and risk factors among adolescents. Some researchers [7,8] have argued the reported increase in PIU research might be due to the inclusion of internet gaming disorder (IGD) in Section III (“Emerging Measures and Models”) of the fifth edition of the Diagnostic and Statistical Manual of Mental Disorders (DSM-5) [9] and gaming disorder (GD) in the 11th revision of the International Classification of Diseases (ICD-11) [10].

PIU is considered within the behavioral addiction spectrum as a type of repeated online behavior leading to significant harm, whereby the individual is unable to control their online behaviors. It persists over a significant period, leading to functional impairments and conflicts with relationships, occupation, and/or education, as well as being associated with a number of online compulsive uses, psychosocial factors, and comorbidities [11,12].

In this context, internet users may become overly attached to using various internet applications resulting in detrimental psychosocial consequences [13,14]. A small proportion of users worldwide (approximately 6% according to Cheng and Li (2014) [15]) experience PIU [16], which is differentiated into the general PIU (GPIU) or specific PIU (SPIU) subtypes (e.g., disordered online gaming) [17]. PIU is now being recognized as an umbrella term for SPIU [14,18,19,20,21]. One of the reasons for the differentiation between GPIU and SPIU is the growing prevalence rate of GPIU associated with problematic online behaviors that can affect individuals across their entire lifespan [2,14,22,23]. This is the reason why it is important to study adulthood across the lifespan (as opposed to adolescents and emerging adults), and it is the first gap addressed in the present study. Indeed, at present, there are few cross-cultural studies examining PIU or other addictive behaviors among adult populations (including the elderly population) [24,25].

From a theoretical perspective, the Interaction of Person-Affect-Cognition-Execution (I-PACE) model supports the contemporary understanding of GPIU, and it is also valid for SPIU [26]. The model posits that these problems are developed as a consequence of the interactions between a person’s predisposing variables, affective and cognitive responses to specific stimuli, and executive functions. From the individual’s perspective, psychopathological correlates (e.g., depression and social anxiety) and temperamental features (e.g., high impulsivity) have been associated to several types of SPIU when interactions occur between specific aspects and situations. Moreover, there has been increasing research on impulsive/reactive neural systems. However, evidence regarding this addictive behavior circuit is still needed. Therefore, the present study addresses a second gap by examining the scientific evidence for the I-PACE model concerning the individual components associated with both GPIU and SPIU cross-culturally.

Cross-cultural studies on GPIU and SPIU have reported cross-national comparisons between two countries from different continents [27], with some studies including cross-country comparisons across a range of countries (i.e., ranging from five to eleven countries), most commonly in Europe [28,29,30], Asia [31], or countries from both continents [32,33]. These cross-cultural studies have generally been conducted with adolescent populations (mean age = 15 years) in relation to demographic and psychosocial factors and the overall GPIU prevalence rate [28,29,31]. In Europe, the prevalence rate of GPIU has been estimated to be between 1.2% (i.e., higher in Romania and Greece (1.7%) versus the Netherlands and Iceland (0.8%)) and 4.4% (i.e., higher in Slovenia (5.8%) versus Italy (1.2%)), while the prevalence rate of PIU has been found to be higher among males than females [28,29].

The only three previous cross-country studies with European samples conducted among adults included GPIU’s associations with personality (self-directedness, self-esteem, defense mechanisms, and coping strategies) [32,33] and other psychopathological symptoms (e.g., depression, anxiety, hostility, and phobic anxiety) [30]. The findings showed those with low self-esteem have a higher tendency to self-report PIU [32], the estimated prevalence rates of PIU ranged between 14.3% (Germany) and 54.9% (United Kingdom (UK)), that GPIU was higher among females, and that GPIU was associated with obsessive-compulsive symptoms, somatization, and hostility across both genders [30]. Additionally, GPIU has been associated with borderline personality traits and immature defense mechanisms, and predictors of GPIU comprise dependent, avoidant, narcissistic, histrionic, and antisocial personality traits [33]. A multinational meta-analysis comprising 31 countries found that GPIU was highest in the Middle East (i.e., 10.9%), and lowest in Northern and Western European regions (i.e., 2.6%) using different psychometric scales [15].

To the best of the present authors’ knowledge, there are no previous multinational studies that have included impulsivity in the study of GPIU [34,35], which is why the construct was included in the present study. Only one SPIU cross-cultural study with 14 countries has assessed impulsivity [36] but as a unidimensional construct. This is a limitation because impulsivity is a multifaceted construct [37], and research has shown that specific impulsivity components (but not all) constitute risk factors for GPIU [38] and SPIU [39]. Including impulsivity as a multidimensional construct is the third gap addressed in the present study, which will inform the I-PACE model regarding individuals’ temperamental features.

In light of the aforementioned literature, the primary aim of the present study was to cross-sectionally investigate PIU among adults, comparing PIU and SPIU across 15 countries (Belgium, Finland, Germany, Italy, Spain, France, Switzerland, Hungary, Poland, UK, Norway, Belgium, Peru, Canada, US, and Indonesia). The secondary aims were to (i) compare perceived use of a number of online activities by users from different countries; (ii) estimate the frequency rate of PIU and SPIU across countries; and (iii) identify risk factors for PIU, including psychopathology and personality characteristics.

## 2. Materials and Methods

### 2.1. Participants and Procedures

The present study comprised a convenience sample with similar characteristics from 15 countries to study PIU obtained through the Tech Use Disorders (TUD) project [40]. The recruitment utilized a snowball sampling procedure by the coauthors’ teams by countries, who spread the survey link and QR code within their lectures, academic institutions sites, email networks, and social media accounts during 2015. The total sample initially comprised 5476 participants. Once the survey data were cleaned, removing data from individuals who did not complete it or did not provide the consent, the data from the remaining sample of 5130 participants were analyzed (93.7% of the overall sample).

The survey was translated into eight languages (i.e., German, French, English, Finnish, Spanish, Italian, Polish, and Hungarian) using the translation and back-translation method [41]. The Catholic University of Louvain (UCLouvain) in Belgium was the coordinating center.

Ethical approval was obtained from the ethics committee of the Psychological Sciences Research Institute (IPSY), which was validated by the European Commission (EC). Informed consent was included within the first page of the online survey ensuring anonymity and confidentiality if the user agreed to participate. The inclusion criteria were being at least 18 years old and being fluent in the language of the survey.

### 2.2. Measures

A structured set of questionnaires was administered in an online survey utilizing *Qualtrics* software organized per language, which included questions on sociodemographic information, technology use, online activities, GPIU and SPIU, and scales assessing psychological factors.

#### 2.2.1. Sociodemographic and Technology Use Variables

Gender and age were the main sociodemographic variables assessed, followed by occupation, relationship, and education status. Other questions related to technology use were asked about online activities engaged in (e.g., emailing, video streaming, video gaming, shopping, and social networking) and time spent on the internet for entertainment (in minutes per day for each online activity) [42,43].

#### 2.2.2. Problematic Internet Use and Psychometric Scales

The five-item version of the Compulsive Internet Use Scale (CIUS-5, well-known as the short CIUS) [44] was used to assess both PIU and SPIU. The latter was assessed by replacing the term “internet” with specific online activities (i.e., social networking, gaming, gambling, sex, and shopping). It comprised five items of the original CIUS (i.e., Items 1, 3, 5, 11, and 12) [16] rated on a five-point Likert scale (i.e., from 0 “never” to 4 “very often”), with scores ranging from 0 to 20. Higher scores indicate greater severity of PIU. Participants scoring higher than or equal to 15 were considered as at-risk of PIU (i.e., corresponding to a scoring of at least 3 (=“often”) on each item). The rationale for this scoring was to apply a “conservative” approach to limit over-pathologizing as much as possible by making the endorsement of each item on the scale mandatory for being considered as presenting problematic usage. The scale has shown good validity and reliability (e.g., Cronbach’s alphas (α) = 0.77 [45] and 0.80 [40]), and its measurement invariance (MI) was previously evaluated [46]. In the present study, the Cronbach’s alphas were adequate (ranging from α in Peru = 0.65 to α in UK = 0.84). The same held for the short CIUS for each specific and global online behaviors in each country and overall sample.

#### 2.2.3. Psychometric Tests Related to Psychological Factors

To assess the three related psychopathological symptoms (psychological distress), the Depression, Anxiety, and Stress Scale (DASS-42) [47] was used in its shorter version (DASS-21) [48], which contains 21 items (seven items per dimension). It is scored using a four-point Likert scale ranging from 0 (“Did not apply to me at all”) to 3 (“Applied to me very much, or most of the time”). The overall score ranges from 0 to 63 (i.e., the score per dimension ranges from 0 to 21). The scale has excellent internal consistency (e.g., α = 0.94, 0.87, and 0.91, respectively [43]). In the present study, Cronbach’s alphas were good (ranging from α in Norway = 0.88, 0.70, and 0.85 to α in Canada = 0.99, 0.99, and 0.99, and in the overall sample α = 0.99, 0.98, and 0.98).

Regarding the temperamental features, the impulsivity traits were assessed using the short Impulsive Behavior Scale (S-UPPS-P) [49], which comprises 20 items evaluating five facets of impulsivity (four items per dimension): negative urgency (the tendency to act rashly in reaction to intense negative emotions), positive urgency (the tendency to act rashly in reaction to intense positive emotions), lack of premeditation (the tendency not to take into consideration long-term consequences of actions), lack of perseverance (the tendency to not be able to remain focused on cognitively demanding or boring tasks), and sensation seeking (the tendency to be open to new or nonconventional experiences and to be involved in stimulating or risky activities). It is scored using a four-point Likert scale ranging from 1 (“Strongly disagree”) to 4 (“Strongly agree”), considering reversed items. The overall score ranges from 20 to 80 (i.e., each dimension ranges from 4 to 16), and it has good internal consistency (e.g., α = 0.70–0.84) and test–retest stability. In the present study, the Cronbach’s alphas were generally adequate (ranging from α in Peru = 0.59–0.83 to α in Italy = 0.80–0.86; in the overall sample α = 0.74–0.83).

### 2.3. Statistical Analyses

Descriptive analyses (i.e., means (M) and standard deviations (SDs) for continuous variables and the proportion (*N*) and percentage (%) for categorical variables) were computed for each participating country to determine the sociodemographic characteristics, technology usage patterns, online activities, and the frequency rate of GPIU and SPIU. A set of nonparametric Kruskal–Wallis H analyses was computed to observe if there were significant differences in the short CIUS scores between countries (i.e., medians (Mdn)). The reason for using this nonparametric test was that the data collected violated an assumption (i.e., heterogeneity of the variance). The Cronbach’s alpha coefficients were computed by each scale, country, and the overall sample to test the reliability. Subsequently, a bivariate correlation matrix using Pearson’s *r* was performed with the overall sample to observe the level of association between the scales in pairs. Finally, a set of multiple regression analyses for the overall sample and per country using the introduction method were computed with the predictors extracted from the correlation analyses to explain the GPIU predictors.

## 3. Results

### 3.1. Sociodemographics, Internet Use by Device, and Activity in Europe and Outside Europe

As can be seen in Table 1, the sample sizes from all countries ranged between 68 in Norway and 602 in Finland. There were more female participants in all samples (i.e., ranging from 65.9% in Germany to 80.9% in Spain). The average age was 24.71 years (SD = 8.70), with a range from 18 to 79 years.

The participants tended to be single university students who had completed their secondary education. The samples were more heterogeneous in Hungary, Italy, and Norway. The majority of users owned both smartphones and computers/laptops and spent between two and three hours on online entertainment using computers and between one and six hours using smartphones per day (these maximum and minimum hours refer to times spent using computers/laptops and smartphones in Hungary and Indonesia).

In relation to online activities engaged in by adult users (see Table 2), the most prevalent behaviors on computers/laptops were emailing, video streaming, social networking using Facebook, and shopping (Facebook was more used by older participants, including individuals in Norway, Italy, and Hungary, while Instagram was used more frequently by younger participants, such as in the US or Indonesia). The online gaming frequency varied between countries and was higher in Germany, Finland, and the US, comprising first person shooter (FPS) games, multiplayer online battle arena (MOBA) games, and massively multiplayer online role-playing games (MMORPGs).

### 3.2. Problematic Internet Use and Psychopathology in and Outside of Europe

In Table 3, the average scores of the scales assessing PIU and the psychological scales, their reliability, and the estimated frequency rate of problematic use in the case of the short CIUS are presented for the 15 surveyed countries. For the short CIUS scores, the results were significantly different between countries (PIU: H(14) = 352.54, *p* < 0.001; gaming: H(14) = 389.79, *p* < 0.001; gambling: H(14) = 220.16, *p* < 0.001; social networking: H(14) = 627.67, *p* < 0.001; cybersex: H(14) = 101.44, *p* < 0.001; shopping: H(14) = 515.76, *p* < 0.001). The scores were lower in European countries in comparison with countries outside of Europe, where scores were twice as high in some cases. The lowest scores in terms of PIU were in Southern European countries (i.e., Spain (Mdn = 3) and Italy (Mdn = 4)) and the highest in American countries (i.e., US (Mdn = 8) and Peru (Mdn = 8)). The frequency rate of PIU ranged from 0.7% in Italy to 10.4% in the US.

In relation to the SPIU frequency rates, Spain and Norway included no cases of problematic video gaming use, participants in Poland and Canada had low frequency rates of problematic gaming (0.4% and 0.5%, respectively), while participants in the US and Indonesia had the highest frequency rates of problematic gaming (4% and 4.7%, respectively). There was no recorded frequency rate of problematic online gambling in many countries except in non-European countries, such as Peru and Indonesia (1% and 0.4%, respectively). In Finland, there was no reported case of problematic social networking, and the other estimated frequency rates of problematic social networking ranged from 0.4% to 7.2% in Europe (Poland and the UK, respectively) and 1.5% to 11% outside Europe (Canada and the US, respectively). There was no reported case of problematic cybersex and problematic online shopping in half of the countries. The frequency rates in the remaining countries ranged from 0.1% to 0.11% for problematic online sex (Indonesia and Switzerland/US, respectively) and from 0.2% to 3.1% for problematic online shopping (Belgium and the US, respectively). In Table 4, the bivariate correlation matrix in the overall sample showed significant relationships between online addictive behaviors and psychological measures.

### 3.3. Regression Analysis to Identify Predictors of Problematic Internet Use in and Outside of Europe

The regression analyses in the overall sample and across countries identified the predictors for PIU (see Table 5). The model for the entire sample explained half of the variance (F (13,3624) = 265.02, *p* < 0.001, R2 adjusted = 0.49, Durbin–Watson = 1.26), including the following risk factors, ordered by strength of association (β statistic): problematic social networking, problematic online gaming, lack of perseverance, problematic online shopping, depression, positive urgency, and problematic online sex.

Overall, the PIU predictors (by order of frequency) were problematic social networking (*n* = 15), problematic online gaming (*n* = 12), lack of perseverance (*n* = 7), positive urgency (*n* = 6), problematic online shopping (*n* = 5), depression (*n* = 4), problematic online sex (*n* = 3), problematic online gambling (*n* = 3), lack of premeditation (*n* = 2), sensation seeking (*n* = 2), stress (*n* = 1), and negative urgency (*n* = 1). A number of predictors explained a significant amount of variance in GPIU for the countries, from 26% of the variance (e.g., Switzerland) to 77% (e.g., UK).

In relation to factors that explained GPIU, problematic social networking and problematic gaming were the relevant predictors, except gaming in Spain, Switzerland, and Peru. A higher intensity (β) of problematic social networking was observed in the UK and Norway, while problematic gaming was high in Finland and Poland. The other problematic internet activities only emerged as predictors in a few countries: problematic online shopping was higher in Canada and the US, and problematic online sex was higher in Finland. Problematic online gambling in France and the US was (unexpectedly) a weak protective factor. For psychopathological predictors, depression was a risk factor for PIU in European countries (i.e., Belgium, Finland, Italy, and Germany). For the impulsivity dimensions, lack of perseverance was a risk factor of PIU in Germany, Belgium, Spain, Finland, Canada, France, and Indonesia. Similarly, positive urgency was a risk factor for PIU in the UK, Peru, Canada, Germany, and Poland, although in Hungary it was (unexpectedly) a weak protective factor. Negative urgency was a protective factor for PIU in Canada. Sensation seeking was a risk factor for PIU in Belgium and a protective factor in the US. Lack of premeditation was unexpectedly a protective factor for PIU in Indonesia.

## 4. Discussion

The aim of the present study was to cross-culturally investigate PIU among adults in 15 countries from European, American, and Asian continents. The specific aims were to compare the perceived engagement in a number of online activities to estimate the frequency rate of GPIU and SPIU and to identify technological, psychopathological, and temperamental risk factors for PIU.

The findings showed that internet use and online activities among adults appeared to be relatively homogeneous across intercontinental nations. The main online entertainment activities on the devices studied was of a social type (i.e., emailing, texting, and social networking) followed by other individual-type entertainment, such as video streaming of music (e.g., Spotify) and watching series or movies (e.g., YouTube and Netflix), which concurs with Laconi et al.’s findings [30]. Differences emerged in which kinds of online social networks were used, and these appeared to depend on age (i.e., Instagram use among younger individuals and Facebook use among older individuals). Furthermore, there were gender differences in online social network use. According to Laor [50], females use Facebook and Instagram more often and are more interactive users than males, who tend to use Twitter. Similarly, younger and single individuals use Instagram more frequently in comparison to older people and couples, which is consistent with the findings in the present study. However, online gaming using computers/laptops in the countries studied was variable in quantity and type because the main genres played included FPS games, MOBA games, and MMORPGs, which share some characteristics regarding their internationalization (e.g., players come from all over the world), complexity (e.g., ranging from lower to higher levels of difficulty), and the need to be connected to play regularly (e.g., to be able to compete) [51].

Compared to the multinational meta-analysis of European users with PIU [15] and the three cross-cultural studies with adult samples [30,32,33], there were fewer at-risk online users in the present study (3.7%; mean CIUS5 = 5.75 out of 20) in comparison to Laconi et al.’s [30] study (6%; mean PIUQ = 17.85 out of 45). The reasons for this discrepancy in results beyond methodological issues were probably due to the fact that most of the epidemiological studies included in the meta-analysis were based on high school students’ self-reports, who usually have more free time relative to adults [52]. Adolescence is known as a period in the lifespan, which is characterized by poorer regulation skills and a proneness to engage more intensively in rewarding and/or risky activities.

Researchers have begun to study whether there is a developmental problem that may impact PIU symptomatology, where personal trajectories may play a role, or whether mental health, academic, social, familial, and interpersonal issues impact PIU development and maintenance [52,53]. Moreover, age in the present sample was slightly higher compared to the previous adult cross-cultural studies [30,32,33]. In fact, some of the present findings may be explained by age rather than cultural background (e.g., sensation seeking was a protective factor in Belgium (M_age_ = 27 years), whereas it was a risk factor in the US (M_age_ = 18 years)), but the different nature of the factor can potentially be explained by age, because older-aged adolescents and emerging adults tend to take more risks compared to younger adolescents and older-aged adults [54].

The PIU frequency rate ranged between 0.7% (Italy) and 10.4% (US), which is lower than the values reported by Laconi et al.’s cross-cultural studies with adults [30,33]. Both the present study and Laconi et al.’s study [30] found that English participants scored higher than participants in other countries (in the present study, participants from both Anglo-Saxon countries (the UK and the US) scored higher than participants in other countries). Interestingly, the US participants had the highest frequency rate of problematic internet use (10.4%). It is noteworthy that in Laconi et al.’s study [30], a different short psychometric scale was used (i.e., the short form of the Problematic Internet Use Questionnaire [55]).

Furthermore, the PIU frequency rate outside of Europe was higher in the present study, as Sariyska et al. [32] and Laconi et al. [33] also reported. In both cases, the frequency rate of PIU was highest in Asian countries. In Sariyska et al.’s study [32], 15.8% in Germany and 18.25% in Spain vs. 47.4% and 64.5% in both samples from China. In Laconi et al.’s study [33], it was 16.68% in France and 17.87% in Finland vs. 23.94% in Iran and 26.98% in the United Arab Emirates. Sariyska et al.’s study [32] used the Internet Addiction Test [56] to assess PIU and obtained higher scores among all European and non-European countries compared to the rates in the present study (e.g., 2.1% in Spain and Germany). It is noteworthy that the present findings concur with recent reviews in which the PIU frequency rate outside of Europe ranged between 13% and 68% [57] or between 36% and 75% [33], which were higher than in European countries, ranging between 4% and 10% among community samples [58] or between 20.5% and 38.4% [33]. Indeed, the present study is the only cross-cultural study that has investigated several SPIUs as well as GPIU.

The online problematic gaming frequency rate in the present study was twice as high outside Europe compared to in Europe (2.6% outside Europe vs. 1% in Europe), as has been reported in previous studies in which there were 5.1% problematic online gamers in Europe [59] and 15% in Peru [60]. This probably explains why the European Parliament has proposed strategies to prevent PIU and SPIU, as identified in the present study, in which problematic gaming and social networking were both identified as being behaviors that need prevention initiatives [57,61].

In addition, a higher frequency rate of problematic social networking has been reported outside of Europe. According to the present study’s results, after PIU, the highest frequency rate of problematic online use was related to problematic social networking, similar to the findings presented in a recent review of GPIU outside Europe [56]. There was a high association between GPIU and problematic social networking, because both overlap in several online activities (e.g., online forums and games). PIU covers all online activities (macrolevel), whereas social networks have narrower online boundaries, i.e., they are specific designed online environments [62]. Therefore, PIU and problematic social networking are heterogenous and can include multiple activities (such as communication, gaming, and gambling). These may be reasons why these online activities obtained the highest frequency rates of problematic use in the countries studied. Contrary to this, problematic online gambling and problematic online sex were almost nonexistent in the present study. It cannot be excluded that these two specific activities tend to be more stigmatized in many jurisdictions and might have influenced the reported frequency rates.

Concerning psychopathological symptoms, a review by Tran et al. (2020) [6] noted that depression was another key predictor of GPIU among adolescents and undergraduate students. The present study also provides evidence regarding this psychopathological correlate as a predictor of GPIU among adult populations, because it has been demonstrated in four of the European countries studied. Interestingly, anxiety and stress were not predictors of GPIU among the adult population in the present study, which seems theoretically logical because an adult usually manages both conditions better than adolescents and emerging adults. Furthermore, in adult population cross-cultural studies, Laconi et al.’s study [30] found anxiety was only a GPIU predictor among males with high PIU scores, explaining that they might use the Internet in order to relieve stress due to the fact of anxiety, while females might use the Internet for other purposes. In the present study, females were more prevalent than males, and this gender imbalance may have impacted the findings.

Regarding the temperamental feature of the multidimensional impulsivity facets, lack of perseverance emerged, which usually relates to poorer attentional skills and procrastination tendencies [63], which was consistently associated with GPIU in both present and past research [38]. Similarly, positive urgency also emerged as a predictor of GPIU in the present study, which can be explained by the fact that positive and negative urgency can be considered a single coherent construct [64] due them both being emotion-related impulsivity traits closely related to each other that can be merged and considered together as “general urgency”. Other impulsivity dimensions were found in the present study, although given the relatively low predictive power of impulsivity traits in the present study, the related findings have to be interpreted with some caution.

### 4.1. Limitations

The limitations included the participant recruitment, with nonprobabilistic samples collecting online self-reported data. This is the reason why the term “frequency rate” was used instead of prevalence throughout the paper. Moreover, national samples had different sample sizes and tended to comprise more young female adults than males. This may explain the relatively high use of the Internet, in general, and of social networking [62] and that females tended to play role-playing games [45]. The frequency rates of problematic use were computed based on those who perceived themselves as usually feeling the symptoms present in the short CIUS, which are the most severe regarding PIU. The present study avoided entering the current debate on the cut-off points [65] given that the approach taken was more conservative than the previous one proposed by Besser et al. [44]. Finally, as seen in other cross-cultural studies among adult populations [30,31,33], it takes a few years to manage all the data and prepare the manuscript, but the present study still provides rich and valuable information and knowledge concerning PIU forms in an understudied age group [24,25].

### 4.2. Future Research Directions

In the future, more cross-cultural research on GPIU and SPIU is needed with more representative samples to offer better generalizations [66]. There are few such studies exploring PIU among the adult population. In addition, among this age group, the findings from the last decade (2013–2018) [30,32,33], including the present study, are heterogeneous but agree on the relevance of different intercontinental prevalence. These studies generally only cover GPIU and a few psychological variables but have not studied SPIU cross-culturally among adults. There is a lack of longitudinal cross-cultural studies. Other biopsychosocial factors may be associated with these online use problems when using constantly evolving technologies (e.g., handheld devices and apps). There is also a lack of coverage for child and elderly populations and other specific population groups (e.g., females or African citizens). Finally, there is need to test and develop further theoretical models (such as the I-PACE model) to deepen evidence-based knowledge and to detail the problematic pathways to these excessive online uses and examining other personal factors highlighted within this model, among other nonpersonal factors.

## 5. Conclusions

To date, no study including adults in both European and non-European countries has been conducted exploring internet use and self-reported PIU (both general and specific). In the present study, participants from 15 countries were compared, online use patterns were assessed, GPIU and SPIU frequency rates of problematic use were estimated, and potential risk/protective factors identified. Higher PIU was found in countries outside of Europe, although intra-European differences also emerged. More specifically, in the case of GPIU, the most important risk factors identified were problematic social networking, problematic gaming, lack of perseverance, and depression. Interestingly, it appears the self-reported risk for PIU by adults was generally low across the samples studied.

## Figures and Tables

**Table 1:** Sociodemographic variables in each country.

	Overall Sample	Germany	Belgium	Spain	Finland	France	Hungary	Italy
***N***	5130	490	586	194	602	457	335	380
**Women (*n* (%))**	3527(69)	3236(5.9)	427 (74.8)	157(80.9)	398(66.1)	375(82.1)	220(65.7)	261(68.7)
**Age (years; *M (SD*))**	24.71(8.70)	25.38(7.06)	26.93(11.88)	25.7(9.07)	27.98(8.65)	25.08(10.24)	27.8(9.13)	28.49(9.83)
**Occupation Status—Student (*n* (*%*))**	3565(69.5)	356(79.5)	387(71.8)	115(68)	457(85.1)	319(78.6)	165(50.3)	186(53.6)
**Civil Status—Single (*n (%))***	2381(55.2)	210(46.9)	314(58.3)	89(52.7)	170(31.7)	252(62.1)	141(43)	229(66)
**Education Status—Secondary Ed. (*n (%))***	440(85.1)	249(55.6)	245(45.5)	46(27.2)	252(46.9)	74(18.2)	104(31.7)	150(43.2)
	**Norway**	**Poland**	**UK**	**Switzerland**	**Canada**	**US**	**Indonesia**	**Peru**
***N***	68	277	98	142	227	356	723	195
**Women (*n (%))***	50(73.5)	201(72.6)	75(76.5)	92(64.8)	156(68.7)	197(55.3)	474(65.6)	121(62.1)
**Age (years; *M (SD*))**	30.69(11.16)	25.17(6.9)	24.84(9.91)	25.39(6.88)	21.8(3.02)	18.89(1.67)	19.28(1.21)	22.66(7.28)
**Occupation Status—Student (*n (%)*)**	39(67.2)	170(64.2)	66(80.5)	101(84.3)	221(97.36)	348(97.8)	720(99.9)	130(86.7)
**Civil Status—Single (*n (%))***	24(41.4)	116(43.8)	54(65.9)	80(66.1)	111(49.3)	318(89.3)	611(84.7)	112(74.7)
**Education Status—Secondary Ed. (*n (%))***	12(20.7)	153(57.7)	42(51.2)	50(41.3)	222(98.2)	146(41)	542(75.2)	43(28.7)

*N* = sample size; *M* = mean; *SD* = standard deviation; *n* = frequency; *%* = valid percentages.

**Table 2:** Online usage patterns per country.

	Overall	Germany	Belgium	Spain	Finland	France	Hungary	Italy
***N***	5130	490	586	194	602	457	335	380
**Computer owner (*n (%*))**	4643(90.5)	441(98.4)	524(97.2)	164(97)	533(99.3)	395(97.3)	316(96.3)	331(95.4)
**Mean (min/day) on computer (*M* (*SD*))**	129.23(173.64)	173.46(173.17)	185.05(188.18)	111.95(257.16)	158.79(176.34)	185.81(173.46)	209.31(187.27)	89.7(114.36)
**Emailing (*n* (*%*))**	4115(80.2)	395(80.6)	484(82.6)	144(74.2)	485(80.6)	359(78.6)	296(88.4)	272(71.6)
**Video streaming (*n (%))***	3403(66.3)	321(65.5)	399(68.1)	87(44.8)	379(63)	297(65)	221(66)	196(51.6)
**Gaming FPS (*n* (*%)*)**	370(7.2)	51(10.4)	21(3.6)	2(1)	67(11.1)	23(5)	14(4.2)	10(2.6)
**Gaming MOBA (*n (%*))**	315(6.1)	36(7.3)	21(3.6)	2(1)	38(6.3)	25(5.5)	22(6.6)	6(1.6)
**Gaming MMORPG (*n (%)*)**	292(5.7)	34(6.9)	34(5.8)	2(1)	47(7.8)	28(6.1)	24(7.2)	11(2.9)
**Buying (*n (%*))**	1825(35.6)	254(51.8)	195(33.3)	34(17.5)	210(34.9)	204(44.6)	112(33.4)	125(32.9)
**Facebook (*n (%)*)**	3436(67)	299(61)	441(75.3)	137(70.6)	424(70.4)	296(64.8)	271(80.9)	249(65.5)
**Instagram (*n (%)*)**	888(17.3)	21(4.3)	44(7.5)	46(23.7)	71(11.8)	40(8.8)	30(9)	53(13.9)
	**Norway**	**Poland**	**UK**	**Switzerland**	**Canada**	**US**	**Indonesia**	**Peru**
***N***	68	277	98	142	227	356	723	195
**Computer owner (*n (%)*)**	58(100)	260(98.1)	81(98.8)	119(98.3)	221(99.5)	348(98)	714(99)	138(92)
**Mean (min/day) on computer (*M* (*SD*))**	130.64(119.45)	188.73(230.91)	230.62(248.67)	159.51(141.7)	105.27(90.45)	174.83(168.43)	191.87(209.78)	189.55(170.89)
**Emailing (*n (%))***	53(77.9)	249(89.9)	69(70.4)	112(78.9)	212(93.4)	292(82)	575(79.5)	118(60.5)
**Video streaming (*n (%*))**	40(58.8)	175(63.2)	60(61.2)	99(69.7)	178(78.4)	281(78.9)	583(80.6)	87(44.6)
**Gaming FPS (*n (%)*)**	1(1.5)	23(8.3)	9(9.2)	7(4.9)	9(4)	48(13.5)	75(10.4)	10(5.1)
**Gaming MOBA (*n (%*))**	0	13(4.7)	3(3.1)	7(4.9)	8(3.5)	33(9.3)	89(12.3)	12(6.2)
**Gaming MMORPG (*n (%)*)**	4(5.9)	6(2.2)	5(5.1)	6(4.2)	1(0.4)	23(6.5)	58(8)	9(4.6)
**Buying (*n (%*))**	27(39.7)	115(41.5)	51(52)	53(37.3)	96(42.3)	186(52.2)	137(18.9)	26(13.3)
**Facebook (*n (%*))**	51(75)	227(81.9)	66(67.3)	72(50.7)	211(93)	245(68.8)	320(44.3)	127(65.1)
**Instagram (*n (%)*)**	16(23.5)	27(9.7)	66(67.3)	9(6.3)	50(22)	161(45.2)	269(37.2)	33(16.9)

*N* = sample size; *M* = mean; *SD* = standard deviation; *n* = frequency; *%* = valid percentages; FPS = first person shooter; MOBA = multiplayer online battle arena; MMORPG = massively multiplayer online role-playing game.

**Table 3:** Descriptive statistics and reliability of problematic internet use and psychopathology and personality measures from fifteen countries.

	Germany	Belgium	Spain	Finland	France	Hungary	Italy	Norway	Poland	UK	Switzerland	Canada	US	Indonesia	Peru
**CIUS-5 Original *M (SD*),** α, Fr	5.37(3.73) 0.77, 2.1	7.00(4.14) 0.74, 5.4	4.14(3.54) 0.71, 2.1	4.98(3.62) 0.75, 1.1	6.71(4.20) 0.72, 5.4	5.67(3.99) 0.77, 3.2	4.62(3.34) 0.66, 0.7	5.74(3.42) 0.71, 2.3	5.74(3.51) 0.71, 1.6	6.49(4.97) 0.84, 10.1	6.82(4.13) 0.69, 6.9	5.38(3.98) 0.78, 2.9	8.01(4.67) 0.78, 10.4	7.66(4.15) 0.73, 7.6	8.04(4.05) 0.65, 6.3
**CIUS-5 Gaming *M (SD*),** α, Fr	1.91(3.41) 0.77, 0.8	2.09(3.61) 0.74, 0.9	0.63(1.61) 0.71, 0	2.29(3.68) 0.75, 1.6	2.22(3.77) 0.72, 1.0	2.02(3.72) 0.76, 1.3	1.55(3.01) 0.66, 0.7	1.30(2.74) 0.71, 0	1.13(2.68) 0.71, 0.4	2.83(4.38) 0.84, 1.4	2.48(4.08) 0.69, 1.1	1.15(2.74) 0.77, 0.5	3.81(4.81) 0.78, 4.0	4.59(4.96) 0.73, 4.7	2.05(3.58) 0.64, 1.0
**CIUS-5 Gambling *M (SD*),** α, Fr	0.06(0.44) 0.86, 0	.09(.64) 0.84, 0	0.17(0.78) 0.63, 0	0.27(1.08) 0.87, 0	0.14(0.98) 0.84, 0	0.29(1.31) 0.88, 0	0.28(1.05) 0.83, 0	0.07(0.34) 0.86, 0	0.17(0.79) 0.87, 0	0.55(1.87) 0.90, 0	0.38(1.68) 0.89, 0	.10(.57) 0.86, 0	0.93(2.42) 0.89, 0	1.05(2.69) 0.87, 0.4	0.81(2.41) 0.83, 1.0
**CIUS-5 Social networking** ***M (SD*),** α, Fr	3.60(3.66) 0.80, 1.0	6.02(4.67) 0.81, 5.6	5.05(4.09) 0.77, 2.8	3.31(3.38) 0.79, 0	4.57(4.60) 0.82, 3.8	4.50(4.07) 0.80, 2.2	4.50(3.88) 0.75, 2.2	5.12(3.75) 0.77, 0	4.74(3.89) 0.77, 0.4	6.42(4.59) 0.82, 7.2	4.21(4.45) 0.83, 3.4	5.32(3.99) 0.77, 1.5	7.98(4.85) 0.79, 11.0	8.26(4.60) 0.76, 10.8	7.99(4.48) 0.75, 9.4
**CIUS-5 Sex ** ***M (SD*),** α, Fr	0.45(1.57) 0.76, 0	.46(1.60) 0.75, 0	0.35(1.54) 0.78, 0	0.44(1.39) 0.69, 0	0.57(1.79) 0.71, 0.3	0.46(1.67) 0.82, 0.3	0.39(1.29) 0.70, 0	0.46(1.44) 0.80, 0	0.30(1.28) 0.78, 0	0.62(2.01) 0.77, 0	1.06(2.77) 0.86, 1.1	0.31(1.34) 0.82, 0	1.36(3.08) 0.85, 1.1	0.93(2.38) 0.79, 0.1	0.67(1.61) 0.62, 0
**CIUS-5 Shopping *M (SD*),** α, Fr	1.68(2.17) 0.68, 0.3	1.40(2.31) 0.70, 0.2	0.91(1.81) 0.67, 0	0.90(1.73) 0.68, 0	2.13(2.75) 0.69, 0	0.87(1.92) 0.72, 0	1.36(2.05) 0.63, 0	1.51(2.21) 0.69, 0	1.41(1.97) 0.64, 0	3.26(3.77) 0.82, 1.4	1.75(3.05) 0.78, 1.1	1.83(2.82) 0.78, 0	4.84(4.24) 0.80, 3.1	3.06(3.87) 0.84, 1.8	0.95(2.02) 0.72, 0
**DASS-21 Stress *M (SD*),** α	4.84(3.66) 0.83	6.43(4.56) 0.85	6.25(4.74) 0.86	4.92(3.68) 0.83	6.57(4.95) 0.84	5.91(4.20) 0.85	6.47(4.54) 0.86	4.68(4.14) 0.85	5.79(4.25) 0.84	5.17(4.71) 0.89	5.61(4.70) 0.85	5.08(4.50) 0.99	5.29(4.45) 0.85	5.90(4.13) 0.85	7.42(4.32) 0.85
**DASS-21 Anxiety *M (SD*),** α	2.47(2.78) 0.74	4.08(3.88) 0.79	3.44(3.92) 0.85	2.93(3.11) 0.78	4.12(4.11) 0.79	3.34(3.35) 0.80	4.64(3.96) 0.80	2.23(2.71) 0.70	3.25(3.68) 0.82	3.34(3.82) 0.83	3.29(3.84) 0.78	2.85(3.86) 0.99	4.15(4.20) 0.83	6.03(4.09) 0.82	4.78(4.25) 0.81
**DASS-21 Depression** ***M (SD*),** α	3.73(3.93) 0.90	4.29(4.41) 0.88	4.08(5.13) 0.94	4.24(4.23) 0.90	4.95(5.32) 0.91	4.98(4.47) 0.90	5.05(4.57) 0.89	3.95(4.26) 0.88	4.47(4.48) 0.89	4.02(4.65) 0.92	3.99(5.12) 0.93	2.86(4.01) 0.99	3.93(4.57) 0.90	4.72(4.29) 0.88	6.19(5.41) 0.91
**S-UPPS-P Negative urgency *M (SD*),** α	8.56(2.39) 0.76	8.87(2.67) 0.84	9.23(2.87) 0.84	8.90(2.79) 0.83	8.96(2.80) 0.82	8.95(2.92) 0.83	10.06(2.87) 0.85	7.71(2.78) 0.88	9.29(2.99) 0.80	9.78(2.75) 0.85	8.64(2.71) 0.85	7.75(2.65) 0.86	9.87(3.03) 0.83	10.14(2.67) 0.80	9.50(2.35) 0.70
**S-UPPS-P Positive urgency *M (SD*),** α	9.22(2.20) 0.69	10.39(2.50) 0.77	9.22(2.37) 0.69	9.26(2.34) 0.72	10.65(2.57) 0.76	9.82(2.63) 0.74	8.79(2.74) 0.86	8.67(2.30) 0.65	10.23(2.61) 0.67	9.97(2.33) 0.76	9.76(2.52) 0.77	9.56(2.64) 0.80	10.67(2.52) 0.72	11.14(2.27) 0.73	9.82(2.30) 0.59
**S-UPPS-P Lack of premeditation *M (SD*),** α	6.88(2.12) 0.81	7.27(2.27) 0.85	7.07(2.19) 0.72	7.74(2.14) 0.71	7.15(2.19) 0.80	6.83(2.19) 0.82	6.51(2.12) 0.80	6.83(2.40) 0.81	6.37(2.20) 0.82	7.00(2.19) 0.78	6.37(2.24) 0.84	5.97(2.31) 0.67	7.08(2.40) 0.79	7.38(1.96) 0.75	7.25(2.38) 0.83
**S-UPPS-P Lack of perseverance** ***M (SD*),** α	6.65(2.26) 0.86	7.07(2.43) 0.90	6.49(2.43) 0.86	6.83(2.14) 0.81	7.05(2.59) 0.89	7.00(2.18) 0.70	6.54(2.05) 0.84	6.48(2.20) 0.85	10.60(3.06) 0.80	10.64(2.28) 0.82	9.84(2.81) 0.86	9.66(2.97) 0.91	11.96(2.55) 0.80	11.47(2.26) 0.71	10.90(3.03) 0.76
**S-UPPS-P Sensation seeking *M (SD*),** α	9.00(2.64) 0.83	9.55(2.77) 0.85	8.83(2.60) 0.79	9.65(2.45) 0.75	9.95(2.95) 0.87	10.00(2.88) 0.81	9.28(2.85) 0.84	9.83(2.95) 0.85	7.32(2.42) 0.82	7.27(2.16) 0.78	6.65(2.23) 0.84	6.02(2.15) 0.89	7.24(2.46) 0.79	7.21(1.95) 0.75	6.95(2.15) 0.83

*M* = mean; *SD* = standard deviation; α = Cronbach’s alpha; Fr = frequency rate.

**Table 4:** Correlation matrix and global reliability of problematic internet use and psychopathological and personality measures of the overall sample.

	*A*	*r*					
**Scales and Dimensions**		1	2	3	4	5	6
**1. CIUS-5 Internet**	0.75	-					
**2. CIUS-5 Gaming**	0.87	0.38 ***	-				
**3. CIUS-5 Gambling**	0.83	0.20 ***	0.35 ***	-			
**4. CIUS-5 Social networking**	0.81	0.64 ***	0.20 ***	0.24 ***	-		
**5. CIUS-5 Online sex**	0.79	0.23 ***	0.34 ***	0.48 ***	0.19 ***	-	
**6. CIUS-5 Shopping**	0.79	0.41 ***	0.21 ***	0.38 ***	0.44 ***	0.26 ***	-
**7. DASS-21 Stress**	0.98	0.29 ***	0.11 ***	0.14 ***	0.28 ***	0.12 ***	0.15 ***
**8. DASS-21 Anxiety**	0.98	0.31 ***	0.21 ***	0.22 ***	0.31 ***	0.17 ***	0.21 ***
**9. DASS-21 Depression**	0.99	0.31 ***	0.19 ***	0.16 ***	0.22 ***	0.16 ***	0.15 ***
**11. S-UPPS-P Negative Urgency**	0.83	0.24 ***	0.11 ***	0.12 ***	0.29 ***	0.08 ***	0.19 ***
**12. S-UPPS-P Positive Urgency**	0.74	0.28 ***	0.16 ***	0.10 ***	0.30 ***	0.10 ***	0.19 ***
**13. S-UPPS-P Lack of Premeditation**	0.81	0.10 ***	0.06 ***	0.09 ***	0.11**	0.08 ***	0.08 ***
**14. S-UPPS-P Lack of Perseverance**	0.82	0.27 ***	0.17 ***	0.10 ***	0.19 ***	0.11 ***	0.11 ***
**15. S-UPPS-P Sensation Seeking**	0.83	0.11 ***	0.12 ***	0.09 ***	0.16 ***	0.07 ***	0.12 ***

α *= Cronbach’s alpha; r* = correlation. ** *p* < 0.01, *** *p* < 0.001.

**Table 5:** Regression analyses predicting generalized problematic internet use regarding specific addictive online problems and psychopathology from 15 countries (β statistics).

CIUS-5 Predictor	Overall	Germany	Belgium	Spain	Finland	France	Hungary	Italy
Gaming	0.21 ***	0.17 ***	0.16 ***	−0.01	0.39 ***	0.18 **	0.20 ***	0.22 ***
Gambling	−0.08 ***	0.03	−0.00	−0.14	−0.09	−0.14 *	−0.03	−0.01
Social networking	0.48 ***	0.40 ***	0.51 ***	0.46 ***	0.45 **	0.42 ***	0.48 ***	0.63 ***
Online sex	0.03 *	0.12 **	−0.04	0.14	0.01	0.14 *	0.08	−0.03
Shopping	0.11 ***	0.16 ***	0.05	0.14	0.05	0.03	0.07	0.05
Stress	0.01	−0.06	−0.13 *	0.07	−0.00	0.15	0.07	−0.16
Anxiety	−0.03	0.31	0.03	−0.07	−0.00	−0.06	−0.02	0.05
Depression	0.13 ***	0.13 *	0.79 **	0.08	0.17 **	0.08	0.12	0.17 *
Negative urgency	−0.21	−0.03	0.04	0.10	−0.00	−0.07	0.06	0.05
Positive urgency	0.08 ***	0.14 *	0.08	−0.02	−0.02	−0.01	−0.12 *	0.00
Lack of premeditation	−0.06 ***	−0.08	−0.10 *	−0.18	−0.07	−0.07	0.07	−0.03
Lack of perseverance	0.14 ***	0.26 ***	0.19 ***	0.19 *	0.18 ***	0.14 *	0.05	−0.01
Sensation seeking	−0.01	−1.80	0.11 **	0.00	0.00	0.03	−0.01	−0.04
*R*^2^ adjusted	0.49	0.44	0.48	0.40	0.52	0.31	0.41	0.47
*D–W*	1.26	1.82	1.39	1.49	1.44	1.09	1.51	1.35
*F*	265.02 ***	21.98 ***	29.07 ***	7.44 ***	34.34 ***	10.18 ***	16.16 ***	16.09 ***
*T*_min_	0.41	0.45	0.45	0.55	0.42	0.70	0.53	0.39
*VIF* _max_	2.42	2.21	2.71	1.81	2.40	1.44	1.88	2.54
**CIUS-5 Predictors**	**Norway**	**Poland**	**UK**	**Switzerland**	**Canada**	**US**	**Indonesia**	**Peru**
Gaming	0.33 *	0.28 ***	0.22 **	0.18	0.25 ***	0.20 ***	0.23 ***	0.08
Gambling	0.09	−0.08	0.01	−0.04	−0.06	−0.12 *	−0.04	−0.04
Social networking	0.67 ***	0.60 ***	0.70 ***	0.46 **	0.48 ***	0.46 ***	0.44 ***	0.50 ***
Cybersex	−0.22	−0.02	−0.16	0.10	0.15 **	−0.02	0.04	0.02
Shopping	−0.06	0.06	0.18 *	0.20	0.18 **	0.18 ***	0.12 **	0.13
Stress	0.18	0.05	−0.32	0.04	0.03	0.07	0.01	−0.17
Anxiety	0.12	−0.03	0.27	−0.11	−0.07	0.04	0.01	0.10
Depression	−0.16	0.12	0.01	0.14	0.07	0.05	0.07	0.27
Negative urgency	0.09	0.02	−0.04	−0.09	−0.22 **	0.05	0.07	−0.03
Positive urgency	−0.01	0.12 *	0.32 **	−0.00	0.15 *	0.06	0.05	0.29 *
Lack of premeditation	−0.26	−0.03	−0.12	0.10	0.07	−0.03	−0.10 **	0.01
Lack of perseverance	0.14	0.03	−0.03	0.25	0.17 **	0.10	0.09 *	0.12
Sensation seeking	0.06	−0.02	−0.09	0.06	0.03	−0.12 *	−0.00	−0.03
*R*^2^ adjusted	0.63	0.61	0.77	0.26	0.57	0.52	0.43	0.43
*D–W*	1.95	1.58	1.61	1.29	1.56	1.34	1.33	1.23
*F*	5.96 ***	27.72 ***	15.16 ***	0.286 ***	20.11 ***	24.78 ***	38.30 ***	4.84 ***
*T* _min_	0.37	0.58	0.35	0.68	0.48	0.64	0.59	0.49
*VIF* _max_	2.69	1.72	2.85	1.67	2.09	1.93	1.75	2.04

Note: *β =* standardized beta coefficient; *p =* level of significance (*p < 0.05, **p < 0 .01, and ***p < 0.001); CIUS-5 scale = Compulsive Internet Use Scale, in its short version as an outcome variable and in its five specific versions (SPIU) as predictors; DASS-21 = Short Depression, Anxiety, and Stress Scale, with these three dimensions as predictors; UPPS-P Scale = Short Impulsive Behavior Scale, with its five dimensions as predictors; *D–W* = Durbin–Watson statistic; *F* = Snedecor statistic; *T min* = minimum tolerance statistic; *VIF max* = maximum VIF statistic.

## Data Availability

Data sharing is not applicable to this article.
